# Supplementary material for: A prospective, multicenter study of invasive fungal disease caused by molds in children and adults in Chile
Source: PLoS One. 2025 Sep 8;20(9):e0330426. doi: 10.1371/journal.pone.0330426 (PMC12416711; doi:10.1371/journal.pone.0330426)
Supplement: Attachment — Submitted filename: Response to Reviewers Feb 2025.docx [file pone.0330426.s004.docx]

Santiago, February 17th, 2025

Dr. Nosheen Nasir
Academic Editor
PLOS ONE

Dear Dr Nasir.

Thank you for your letter referring to the revision of our manuscript PONE-D-24-19295. We also thank the reviewers for their diligent revision and comments that will certainly improve the manuscript. We will address the reviewer comments one by one, pointing out when changes to the original manuscript were made following the reviewers’ suggestions.

**Reviewers' comments:**
**Reviewer #1:** Invasive mold disease (IMD) is a severe complication which affects immunocompromised and immunocompetent patients. In this manuscript, the authors have investigated the epidemiological and clinical features of IMD in patients specifically from Chile between the time period of 2019 to 2021. Some suggestions regarding improving or modifying the manuscript is as follows:

1. Page 4: Please re write the abstract since it seems rather disconnected and abrupt.

**Response.** We re wrote the manuscript in a more comprehensive manner.

2. Page 4: In the abstract, the words "The objective" is in italics.

**Response.** We delete the italics

3. Page 4: In the results --What is immunosuppression in the pediatric group, the result of?

**Response.** Thanks for this suggestion. We change the term immunosuppression by the following phrase in the abstract: Cancer, neutropenia and hematopoietic cell transplantation were the most frequently associated conditions in the pediatric group, and we added the same information in the Results section (page 12)

4. Page 8: Could you explain in detail what the patient conditions were, for data analysis?

**Response.** The immunosuppression-related conditions were cancer, neutropenia and HCT, as was explained in the abstract and results.

5. The numerical data is not consistent throughout the paper --in some places the numbers are given as digits and in other places, the numbers are spelt out.

**Response.** We change all numbers to digits, with the exception of numbers that are at the beginning of a paragraph or immediately after a period.

6. Please provide the full forms of all the abbreviations mentioned.

**Response.** The full forms were provided previous to abbreviations throughout the manuscript

7. Have you looked into Crytococcosis, considering that WHO has mentioned it as an emerging disease?

**Response.** Thanks for this suggestion. We don’t inform about Cryptococcus because this paper aimed to describe infections caused by molds, not yeasts.

**Reviewer #2:** Report on Review of "A prospective, multicenter study of invasive fungal disease caused by molds in children and adults in Chile" The submitted manuscript offers valuable insights into the epidemiology of IMDs in Chile. However, there are several areas where the manuscript could be improved to enhance clarity and comprehensiveness.

1.Terminology: The authors should consistently use the plural form "Invasive mold diseases (IMDs)" throughout the manuscript, as IMDs encompass a broad spectrum of diseases caused by various mold species.

**Response**. We changed IMD to IMDs throughout the manuscript

2.Study Design: Please clarify the composition of the control group in this prospective observational study. How were the control subjects selected and compared to the case group?

**Response**. This study only included IMDs cases. Controls were not included

3. A detailed description of the inclusion and exclusion criteria for both groups would strengthen the study design.

**Response**. We incorporated a IMD case definition in the Patients and methods section, to better describe the cases included (Page 10)

4. Methodology: A more comprehensive the sample description of collection, processing, and culture techniques is needed.-The rationale for limiting antifungal susceptibility testing to Aspergillus Spp. should be explained.

**Response**. Thanks for these important suggestions. We improve the Laboratory procedures paragraph including collection, culture, identification (Page 9-10), and the explanation that not all the Aspergillus strains were viable for the susceptibility test (Page 17), also we include a commentary that it is a limitation of our study (Page 24)

5. Results: The clinical manifestations of IMDs should be presented separately for each type of infection to provide a clearer understanding of the disease spectrum.

**Response**.

Thanks again for the commentary. We include a new table (Table 3) with more information about the clinical manifestation in aspergillosis, mucormycosis and fusariosis cases, describing in the manuscript the more relevant clinical differences among them (Page 15)

6. The specific molecular technique used for the identification of mold species should be stated.

**Response**. We include these specifications

7. The results for children and adults should be presented separately, especially for the proven, probable, and possible categories.

**Response**. The outcomes for children and adults, in general and for aspergillosis, mucormycosis and fusariosis are detailed in Table 5

8. A more detailed analysis of the antifungal treatment regimens would be beneficial. For each disease, please specify which antifungal agents were used and for what duration.

**Response**. We include these data, in special we include the antifungal therapy for aspergillosis, mucormycosis and fusariosis. (Page 18)

9. Discussion: Although the manuscript centers on IMDs, it disproportionately emphasizes Aspergillus. The data, however, indicates that fusariosis and mucormycosis are prevalent infections among pediatric and adult populations. The discussion should give more weight to these findings and their implications for clinical management.

**Response**. Thanks for this suggestion. We consider that inclusion of clinical manifestations and therapy in the results section (Commentaries 5 & 8), we answer this comment. In addition, we include in the discussion to consider the differential diagnosis of IMD in the clinical approach and therapeutic decision-making process (Page 19-20).

We truly hope that our responses to the reviewers and the modifications made to our manuscript make our paper suitable for publication in your Journal.

Sincerely yours,

Ricardo Rabagliati
