# Supplementary material for: A prospective, multicenter study of invasive fungal disease caused by molds in children and adults in Chile
Source: PLoS One. 2025 Sep 8;20(9):e0330426. doi: 10.1371/journal.pone.0330426 (PMC12416711; doi:10.1371/journal.pone.0330426)
Supplement: Attachment — Submitted filename: review opinion.docx [file pone.0330426.s003.docx]

1.What are the clinical implications of statistically comparing age-related differences between adult and pediatric populations?

2. Based on the statistical results of the length of stay parameter in table 4, the median and interquartile range (IQR)might be the more appropriate choices.
3. why so few of BDG test were performed in the current study？
4. Why there are so many letter “a” in table 3 ?
5. （Page 10 ,line 1^st^ ） Please describe the diagnostic criteria for fungal infections in detail within the text, rather than simply citing references.
